# Retraction Note: Isolation, expression, and in silico profiling of a thermostable xylanase from *Geobacillus stearothermophilus* strain NASA267: insights into structural features and agro-waste valorization

**DOI:** 10.1186/s12934-025-02790-1

**Published:** 2025-07-10

**Authors:** Safaa M. Ali, Nehad Noby, Nadia A. Soliman, Sanaa H. Omar

**Affiliations:** 1 Nucleic Acid Research Department, GEBRI, City of Scientific Research and Technological Applications, Alexandria, Egypt; 2 Department of Biotechnology, Institute of Graduate Studies and Research, Alexandria University, Alexandria, 21526 Egypt; 3 Bioprocess Development Department, GEBRI, City of Scientific Research and Technological Applications, Alexandria, Egypt; 4 Botany and Microbiology Department, Faculty of Science, Alexandria University, Alexandria, Egypt

**Retraction Note: Microbial Cell Factories (2025) 24:69**

10.1186/s12934-025-02672-6

The Editor-in-Chief has retracted this article. After publication, concerns were raised regarding the data presented in some of the figures. Specifically:

1. In Fig. 2, the PCR blot images, before and after purification, appear to overlap.
2. Figure 5 appears to overlap with Fig. 1 in [1].
3. Figure 6 appears to overlap with Fig. 9 in [2].

The Editor-in-Chief has therefore lost confidence in the reliability of the results and conclusions presented.

Safaa M. Ali disagrees with this retraction, and none of the remaining authors have responded to correspondence from the publisher about this retraction.
